# Exploring the Pathogenesis of Psoriasis Complicated With Atherosclerosis *via* Microarray Data Analysis

**DOI:** 10.3389/fimmu.2021.667690

**Published:** 2021-05-27

**Authors:** Wenxing Su, Ying Zhao, Yuqian Wei, Xiaoyan Zhang, Jiang Ji, Shun Yang

**Affiliations:** ^1^ Department of Plastic and Burn Surgery, The Second Affiliated Hospital of Chengdu Medical College, China National Nuclear Corporation 416 Hospital, Chengdu, China; ^2^ Department of Dermatology, The Second Affiliated Hospital of Soochow University, Suzhou, China

**Keywords:** psoriasis, atherosclerosis, bioinformatics, differentially expressed genes, hub genes

## Abstract

**Background:**

Although more and more evidence has supported psoriasis is prone to atherosclerosis, the common mechanism of its occurrence is still not fully elucidated. The purpose of this study is to further explore the molecular mechanism of the occurrence of this complication.

**Methods:**

The gene expression profiles of psoriasis (GSE30999) and atherosclerosis (GSE28829) were downloaded from the Gene Expression Omnibus (GEO) database. After identifying the common differentially expressed genes (DEGs) of psoriasis and atherosclerosis, three kinds of analyses were performed, namely functional annotation, protein‐protein interaction (PPI) network and module construction, and hub gene identification and co-expression analysis.

**Results:**

A total of 94 common DEGs (24 downregulated genes and 70 upregulated genes) was selected for subsequent analyses. Functional analysis emphasizes the important role of chemokines and cytokines in these two diseases. In addition, lipopolysaccharide-mediated signaling pathway is closely related to both. Finally, 16 important hub genes were identified using cytoHubba, including LYN, CSF2RB, IL1RN, RAC2, CCL5, IRF8, C1QB, MMP9, PLEK, PTPRC, FYB, BCL2A1, LCP2, CD53, NCF2 and TLR2.

**Conclusions:**

Our study reveals the common pathogenesis of psoriasis and atherosclerosis. These common pathways and hub genes may provide new ideas for further mechanism research.

## Introduction

More and more evidence show that psoriasis increases the relative risk of atherosclerosis (about 25%) and is independent of risk factors, such as smoking, obesity, and hyperlipidemia (1). Patients with a course of psoriasis more than 8 years are more likely to develop atherosclerosis than non-psoriatic patients (2). Atherosclerosis is the pathological basis of psoriasis complicated with cardiovascular disease. Atherosclerosis and psoriasis have many overlapping inflammatory environmental factors, including local and systemic immune processes, inflammatory cytokines/chemokines, such as serum TNF-α, vascular endothelial growth factor, IL12, monocyte chemotaxis Protein 1, S100A8/A9 and significantly elevated IL17A, etc. (3).

Although psoriasis is considered as a risk factor for developing atherosclerosis, the exact mechanisms that explain the coexistence of these two disorders remain unclear. The IL23/IL17 axis could be the most fundamental pathway. Psoriasis and atherosclerosis have overlapping mechanisms in pathogenic pathways. In psoriasis, myeloid dendritic cells secrete IL12 and IL23, prompting T cells to differentiate into Th1 and Th17 cell subtypes; Th1 cells secrete TNF-α and IFN-γ, which promote the activation and proliferation of keratinocytes and the expression of adhesion molecules such as intercellular adhesion molecule 1 (4); Th17 cells secrete IL17 and IL22 to promote the proliferation of keratinocytes and angiogenesis in the lesion (5). In atherosclerosis, the activation of endothelial cells in the plaques of neonatal arteries promotes monocyte and lymphocyte overflow, and granulocyte-macrophage colony stimulating factor can promote the synthesis of IL12 and IL23 by macrophages and dendritic cells, reducing BCL2 and activating Th1 and Th17 responses (6). Th1 cells secrete TNF-α and IFN-γ to promote the further growth of atherosclerotic plaques, while Th17 cells secrete IL17 and IL22, and the release of reactive oxygen species increases, which promotes the formation of new blood vessels and intraplaque hemorrhage; Elevated IL17 levels in plaques may further weaken the fibrous caps in atherosclerotic plaques, leading to plaque rupture and myocardial infarction (1, 7).

The common transcription feature may provide new insights into the common pathogenesis of psoriasis and atherosclerosis. The purpose of this study is to identify hub genes related to the pathogenesis of psoriasis complicated with atherosclerosis. We analyzed two gene expression data sets (GSE30999 and GSE28829) downloaded from the GEO database. Comprehensive bioinformatics and enrichment analysis were used to determine the common DEGs and its functions of psoriasis and atherosclerosis. In addition, PPI network was constructed to analyze gene modules and identify hub genes by using STRING database and Cytoscape software. In the end, we identified 16 important hub genes, and we further analyzed the transcription factors of these genes and verified their expression. The hub genes identified here between psoriasis and atherosclerosis are expected to provide new insights into the biological mechanisms of these two diseases.

## Materials and Methods

### Data Source

GEO (http://www.ncbi.nlm.nih.gov/geo) (8) is a public database containing a large number of high-throughput sequencing and microarray data sets submitted by research institutes worldwide. We searched for related gene expression datasets using psoriasis and atherosclerosis as keywords. The inclusion criteria are set as: two independent expression profiles come from the same sequencing platform and contain the largest sample size. In addition, the test specimens included should be from humans. finally, two microarray datasets [GSE30999 (9) and GSE28829 (10)] were downloaded from it (Affymetrix GPL570 platform, Affymetrix Human Genome U133 Plus 2.0 Array). The GSE30999 dataset contains 85 paired psoriasis patients with skin lesions (LS) and adjacent normal tissues (NL). GSE28829 consists of 13 early atherosclerotic plaque samples (EA) and 16 advanced atherosclerotic plaque samples (AA) from the human carotid artery.

### Identification of DEGs

GEO2R (www.ncbi.nlm.nih.gov/geo/ge2r) (11) is an online analysis tool developed based on two R packages (GEOquery and Limma). The GEOquery package is used to read data, and the Limma package is used to calculate the differential expression multiple. We used GEO2R to compare gene expression profiles between different groups to determine the DEGs between the diseased group and the control group. Probe sets with no corresponding gene symbols or genes with more than one probe set were removed or averaged, respectively. Only the genes with adjusted P-value < 0.05 and |logFC (fold change) | ≥ 1 were identified as DEGs. The online Venn diagram tool was used to obtain their common DEGs.

### Enrichment Analyses of DEGs

Gene ontology (GO) is a database established by the Gene Ontology Federation, which provides simple annotations of gene products from functions, biological pathways involved, and location in cells. Kyoto Encyclopedia of Genes and Genomes (KEGG) Pathway is a database dedicated to storing information on gene pathways in different species. KEGG Orthology Based Annotation System (KOBAS) (http://kobas.cbi.pku.edu.cn) (12) is a Web server for gene/protein functional annotation and functional enrichment developed by Peking University, which collects 4325 species functional annotation information. The enrichment analysis results of GO and KEGG Pathway were obtained from the KOBAS 3.0 database. Adjusted P-value < 0.05 was considered significant.

### PPI Network Construction and Module Analysis

Search Tool for the Retrieval of Interacting Genes (STRING; http://string-db.org) (version 11.0) (13) can search for the relationship between proteins of interest, such as direct binding relationships, or coexisting upstream and downstream regulatory pathways, to construct a PPI network with complex regulatory relationships. Interactions with a combined score over 0.4 were considered statistically significant. Cytoscape (http://www.cytoscape.org) (version 3.7.2) (14) was used to visualize this PPI network. Cytoscape’s plug-in molecular complex detection technology (MCODE) was used to analyze key functional modules. Set the selection criteria as: K-core = 2, degree cutoff = 2, max depth = 100, and node score cutoff = 0.2. Then the KEGG and GO analysis of the involved modular genes were performed with KOBAS 3.0.

### Selection and Analysis of Hub Genes

The hub genes were identified by using the cytoHubba plug-in of Cytoscape. Here, we used seven common algorithms (MCC, MNC, Degree, Closeness, Radiality, Stress, EPC) to evaluate and select hub genes. Subsequently, we constructed a co-expression network of these hub genes *via* GeneMANIA (http://www.genemania.org/) (15), which is a reliable tool for identifying internal associations in gene sets.

### Validation of Hub Genes Expression in Other Data Sets

The mRNA expression of identified hub genes was verified in GSE14905 (16) and GSE100927 (17). The GSE14905 dataset contains 33 LS, 28 NL and 21 normal skin (NS). GSE100927 consists of 69 AA and 35 control artery samples (CA) from the human artery. The comparison between the two sets of data was performed with the T-test. P-value < 0.05 was considered significant.

### Prediction and Verification of Transcription Factors (TFs)

Transcriptional Regulatory Relationships Unraveled by Sentence-based Text mining (TRRUST) (18) is a database for the prediction of transcriptional regulatory networks, which contains the target genes corresponding to TFs and the regulatory relationships between TFs. TRRUST currently includes two species: human and mouse, which contain 8,444 and 6,552 TFs target regulatory relationships of 800 human TFs and 828 mouse TFs, respectively. TFs that regulate the hub genes were obtained through the TRRUST database, and adjusted P-value < 0.05 was considered significant. Subsequently, we verified the expression levels of these TFs in GSE30999 and GSE28829 with the T-test.

## Results

### Identification of DEGs

**Figure 1:**
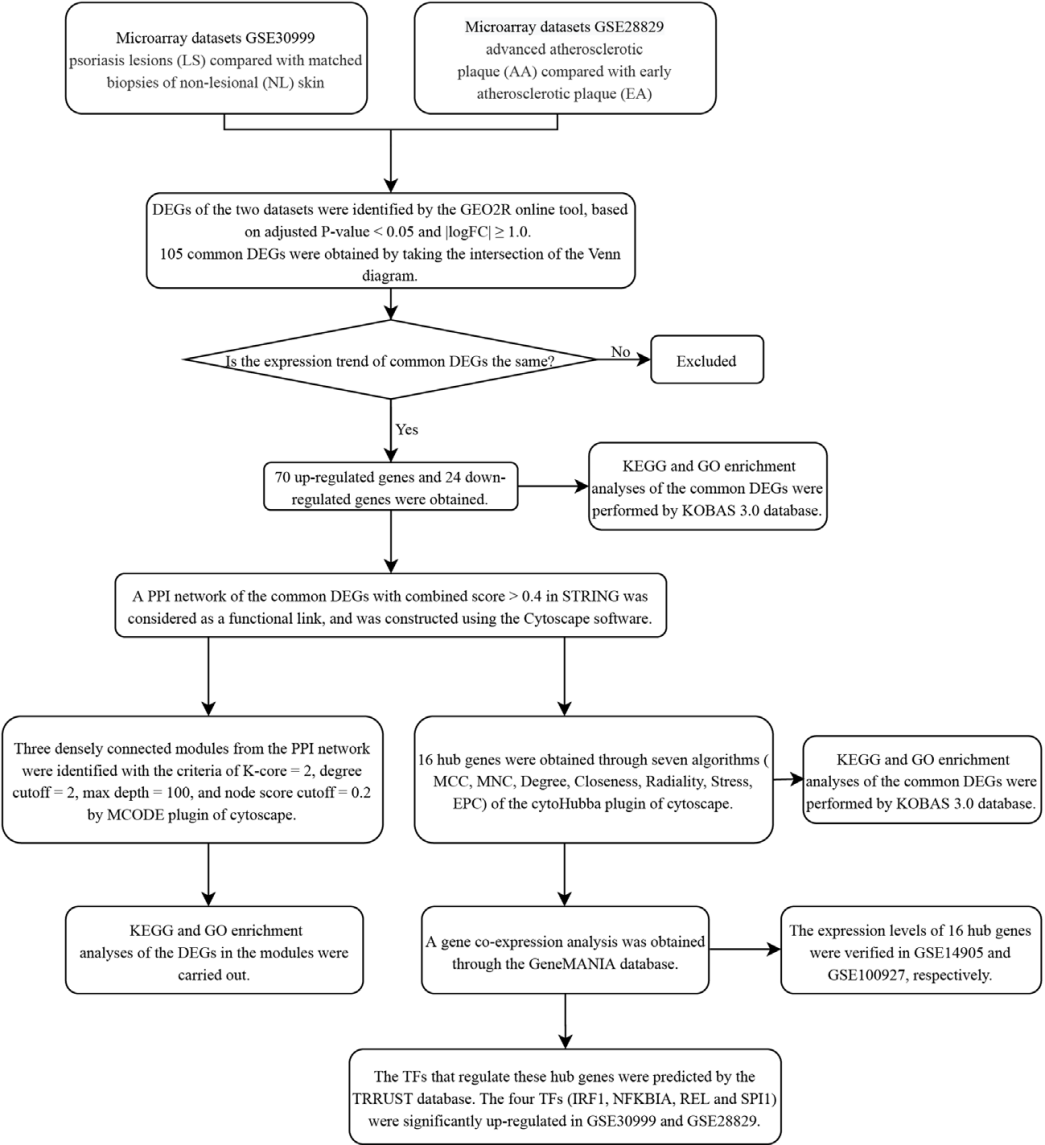
Research design flow chart.

**Figure 2:**
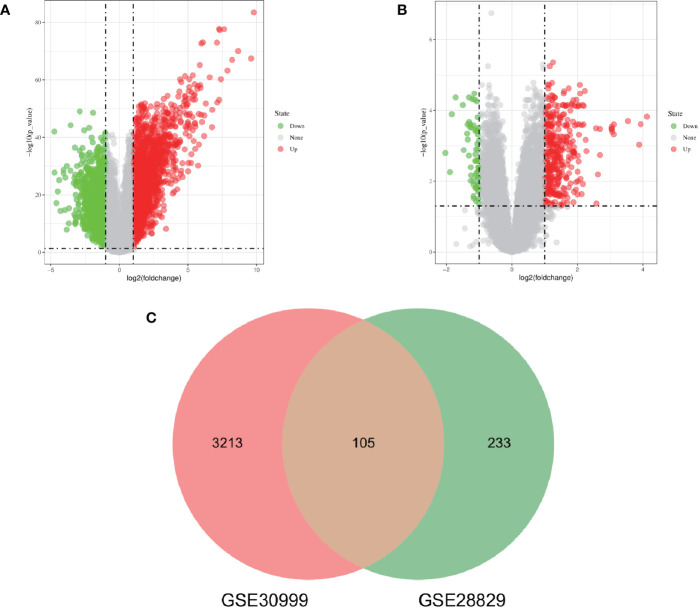
Volcano diagram and Venn diagram. **(A)** The volcano map of GSE30999. **(B)** The volcano map of GSE28829. Upregulated genes are marked in light red; downregulated genes are marked in light green. **(C)** The two datasets showed an overlap of 105 DEGs.

The research flowchart of this research was shown in **Figure 1**. After standardizing the microarray results, DEGs (3318 in GSE30999 and 338 in GSE28829) were identified (**Figures 2A, B**). After taking the intersection of the Venn diagram, 105 common DEGs were obtained (**Figure 2C**). Subsequently, we obtained 94 DEGs (**Table S1**) after excluding genes with opposite expression trends in GSE30999 and GSE28829.

### Analysis of the Functional Characteristics of Common DEGs

**Figure 3:**
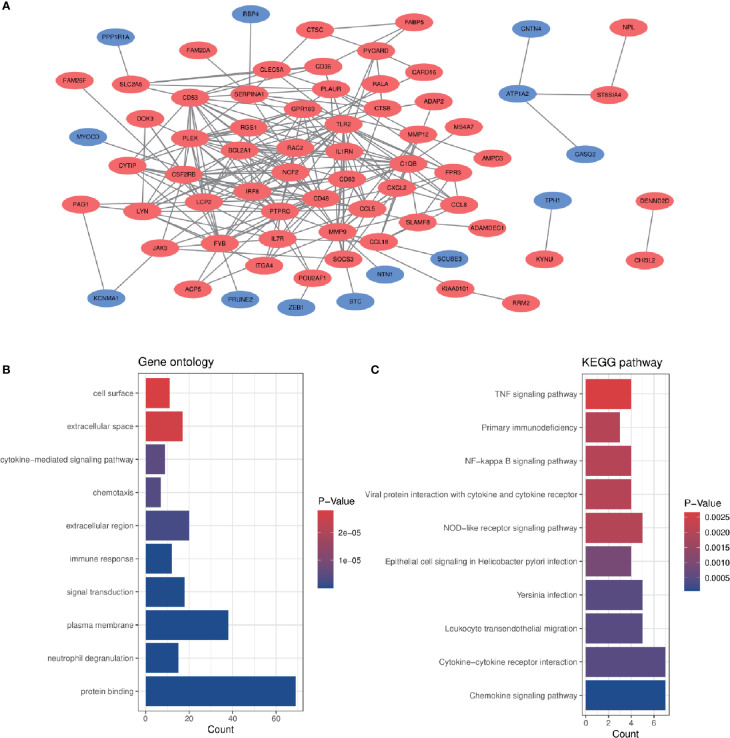
PPI network and common DEGs enrichment analysis results. **(A)** PPI network diagram. Red indicates up-regulated genes, and blue-violet indicates down-regulated genes. **(B, C)** The enrichment analysis results of GO and KEGG Pathway. Adjusted P-value < 0.05 was considered significant.

In order to analyze the biological functions and pathways involved in the 94 common DEGs, GO and KEGG Pathway enrichment analysis were performed. GO analysis results show that these genes were mainly enriched in protein binding (P = 7.11E-15), neutrophil degranulation (P = 5.81E-10), immune response (P = 1.55E-07), chemotaxis (P = 3.91E-06) and cytokine-mediated signaling pathway (P = 5.19E-06) (**Figure 3B**). In terms of KEGG Pathway, the three significant enrichment pathways are chemokine signaling pathway (P = 7.44E-05), cytokine-cytokine receptor interaction (P = 4.68E-04) and leukocyte transendothelial migration (P = 4.68E-04) (**Figure 3C**). These results strongly indicate that chemokines and cytokines are jointly involved in the occurrence and development of these two inflammatory diseases.

### PPI Network Construction and Module Analysis

**Figure 4:**
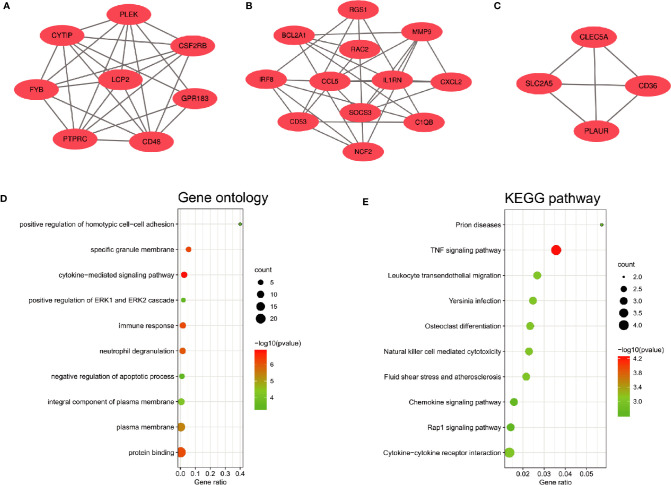
Significant gene module and enrichment analysis of the modular genes **(A–C)** Three significant gene clustering modules. **(D, E)** GO and KEGG enrichment analysis of the modular genes. The size of the circle represents the number of genes involved, and the abscissa represents the frequency of the genes involved in the term total genes.

The PPI network of the common DEGs with combined scores greater than 0.4 was constructed using Cytoscape, which contained 72 nodes and 230 interaction pairs (**Figure 3A**). Three closely connected gene modules were obtained through MCODE plug-in of Cytoscape, including 24 common DEGs and 59 interaction pairs (**Figures 4A–C**). GO analysis showed that these genes are related to inflammation and immune response (**Figure 4D**). KEGG Pathway analysis showed that them to be mainly involved in TNF signaling pathway, cytokine-cytokine receptor interaction and leukocyte transendothelial migration (**Figure 4E**).

### Selection and Analysis of Hub Genes

**Figure 5:**
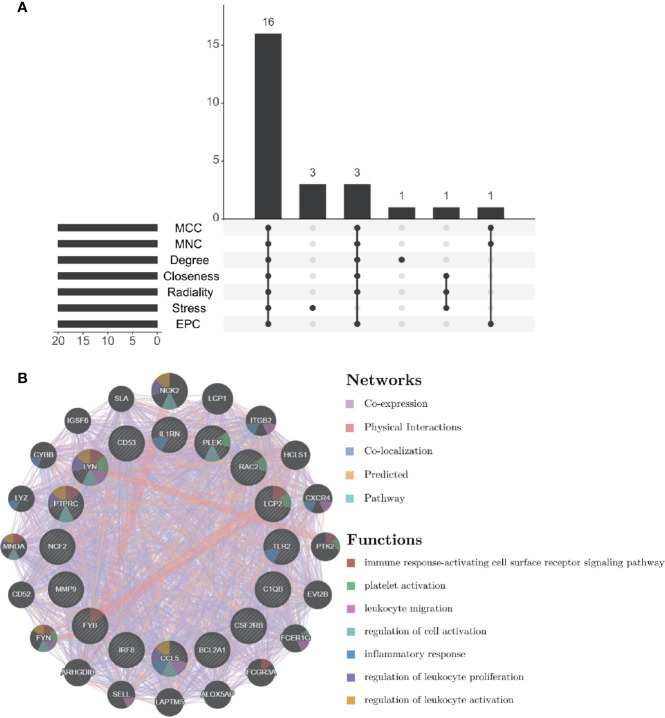
Venn diagram and co-expression network of hub genes. **(A)** The Venn diagram showed that seven algorithms have screened out 16 overlapping hub genes. **(B)** Hub genes and their co-expression genes were analyzed *via* GeneMANIA.

**Figure 6:**
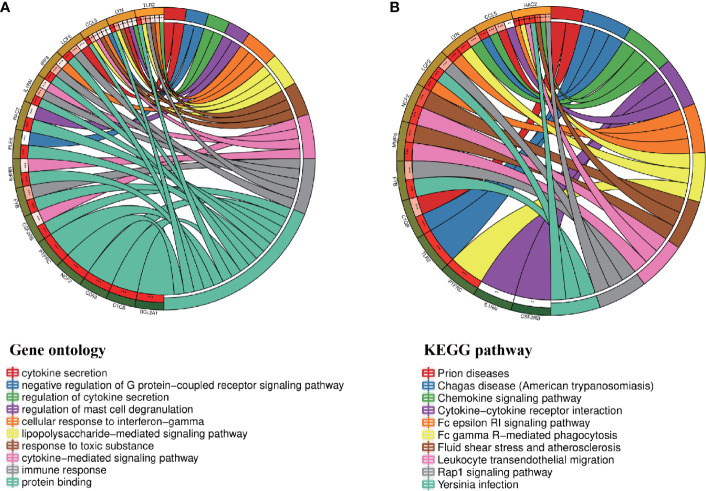
Enrichment analysis of the hub genes **(A, B)** GO and KEGG enrichment analysis of the hub genes. The outermost circle is term on the right and the inner circle on the left represents the significant P-value of the corresponding pathway of the gene.

Through the seven algorithms of plug-in cytoHubba, we have calculated the top 20 hub genes (**Table 1**). After taking the intersection of the Venn diagrams, we found 16 common hub genes, including LYN, CSF2RB, IL1RN, RAC2, CCL5, IRF8, C1QB, MMP9, PLEK, PTPRC, FYB, BCL2A1, LCP2, CD53, NCF2 and TLR2 (**Figure 5A**). **Table 2** shows their full names and related functions. Based on the GeneMANIA database, we analyzed the co-expression network and related functions of these genes. These genes showed the complex PPI network with the co-expression of 80.18%, physical interactions of 11.58%, co-localization of 4.81%, predicted of 2.45% and pathway of 0.99% (**Figure 5B**). GO analysis showed that these genes are mainly involved in protein binding, lipopolysaccharide-mediated signaling pathway, immune response, cytokine-mediated signaling pathway and cellular response to interferon-gamma (**Figure 6A**). These results emphasized the important role of lipopolysaccharide and cytokines in these two diseases. In addition, KEGG Pathway analysis showed that they are mainly involved in Fc epsilon RI signaling pathway, Fc gamma R-mediated phagocytosis, leukocyte transendothelial migration and chemokine signaling pathway. Interestingly, three genes (NCF2, RAC2 and MMP9) were also involved in fluid shear stress and atherosclerosis (**Figure 6B**).

**Table 1:** The top 20 hub genes rank in cytoHubba.

MCC	MNC	Degree	Closeness	Radiality	Stress	EPC
PLEK	PLEK	IL7R	PLAUR	PLAUR	SERPINA1	PLEK
CXCL2	CXCL2	PLEK	PLEK	PLEK	PLAUR	CXCL2
C1QB	C1QB	CXCL2	CXCL2	CXCL2	PLEK	C1QB
CD48	CD48	C1QB	C1QB	C1QB	C1QB	CD48
CCL5	CCL5	CD48	CD48	CD48	CCL5	CCL5
CYTIP	CYTIP	CCL5	CCL5	CCL5	MMP12	CYTIP
CD53	CD53	CD53	CD53	CD53	CD53	CD53
GPR183	GPR183	GPR183	GPR183	GPR183	BCL2A1	GPR183
BCL2A1	BCL2A1	BCL2A1	BCL2A1	BCL2A1	LCP2	BCL2A1
LCP2	LCP2	LCP2	LCP2	LCP2	RAC2	LCP2
RAC2	RAC2	RAC2	RAC2	RAC2	FYB	RAC2
FYB	FYB	FYB	FYB	FYB	IL1RN	FYB
IL1RN	IL1RN	IL1RN	IL1RN	IL1RN	JAK3	IL1RN
IRF8	IRF8	IRF8	IRF8	IRF8	TLR2	IRF8
TLR2	TLR2	TLR2	TLR2	TLR2	IRF8	TLR2
LYN	LYN	LYN	LYN	LYN	LYN	LYN
MMP9	MMP9	MMP9	MMP9	MMP9	MMP9	MMP9
NCF2	NCF2	NCF2	NCF2	NCF2	NCF2	NCF2
PTPRC	PTPRC	PTPRC	PTPRC	PTPRC	PTPRC	PTPRC
CSF2RB	CSF2RB	CSF2RB	CSF2RB	CSF2RB	CSF2RB	CSF2RB

**Table 2:** The details of the hub genes.

No.	Gene symbol	Full name	Function
1	LYN	v-yes-1 Yanaguchi sarcoma viral related oncogene homolog	Belongs to the Src protein kinase family. LYN is involved in cell proliferation, differentiation, apoptosis, migration and Metabolic activity and regulation of cellular immunity.
2	CSF2RB	Colony Stimulating Factor 2 Receptor, Beta	It is the shared subunit of receptors for interleukin 3, colony stimulating factor 2 (CSF2) and IL5, and is responsible for the initiation of signal transduction triggered by ligand binding.
3	IL1RN	interleukin-1-receptor antagonist	IL1RN is a protein that binds to IL-1 receptors and inhibits the binding of IL-1α and IL-1β.
4	RAC2	ras-related C3 botulinum toxin substrate 2	belongs to the Rho family of GTP-binding proteins, Rac2 is only found in cells of myeloid origin, Involves in regulating human monocyte chemotaxis and NADPH oxidase activity.
5	CCL5	chemokine (C-C motif) ligand 5	CCL5 is highly expressed in cancer where it contributes to inflammation and malignant progression.
6	IRF8	Interferon regulatory factor 8	Plays critical roles in interferon (IFN) signaling pathways governing the establishment of innate immune responses by myeloid and dendritic cells.
7	C1QB	complement C1q subcomponent subunit B	Plays an important role in maintaining the stability of the body’s environment, oxidative stress, glucose and lipid metabolism and other processes
8	MMP9	matrix metalloproteinase 9	endoprotease with collagenase and/or gelatinase activity which exert deleterious effects on the endothelium integrity and collagen fibers.
9	PLEK	Pleckstrin homology domain containing	Involves in the apoptosis of vascular endothelial cells.
10	PTPRC	encoding protein-tyrosine phosphatase receptor-type C	PTPRC is one of the most abundant leukocyte cell surface glycoproteins and is expressed exclusively upon cells of the hematopoietic system.
11	FYB	Fyn-binding protein	FYB is a hematopoietic-specific adapter, which associates with and modulates function of SH2-containing leukocyte phosphoprotein of 76 kilodaltons (SLP-76).
12	BCL2A1	B-cell lymphoma-2 related protein A1	BCL2A1 is important cell death regulators, whose main function is to control the release of cytochrome c from mitochondria in the intrinsic apoptotic pathway.
13	LCP2	lymphocyte cytosolic protein 2	Participates in immune response and intracellular signal transduction.
14	CD53	CD53	CD53 has been implicated in B cell development and function. CD53 is a transcriptional target of EBF1, a critical transcription factor for early B cell development.
15	NCF2	neutrophil cytosolic factor 2	NCF2, the gene encoding the NADPH oxidase cytosolic component p67phox, is up-regulated by TNF-α.
16	TLR2	Toll-like receptor 2	TLR-2 triggers receptor-mediated events, including cytokine-mediated inflammation.

### Validation of Hub Genes Expression

**Figure 7:**
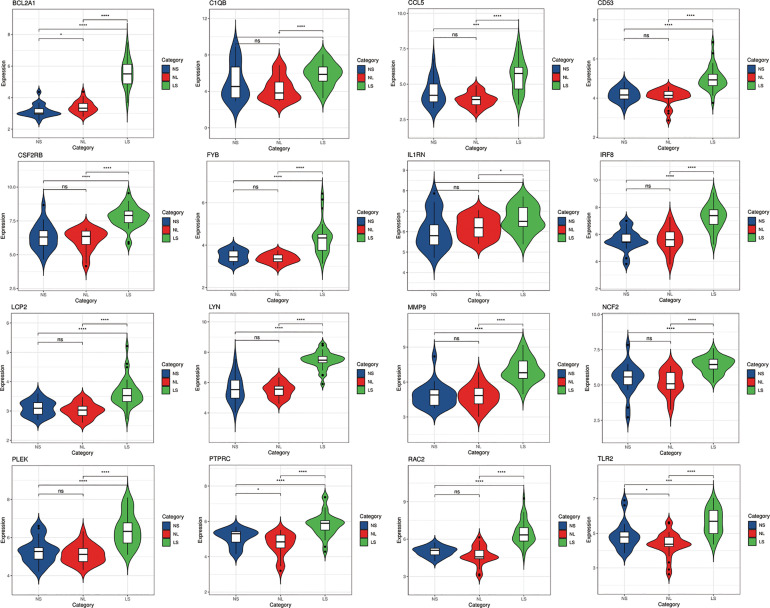
The expression level of hub gene in GSE14905. The comparison between the two sets of data uses the mean T test. P-value < 0.05 was considered statistically significant. LS, skin lesions; NL, adjacent normal tissues; NS, normal skin. *p < 0.05; ***p < 0.001; ****p < 0.0001.

**Figure 8:**
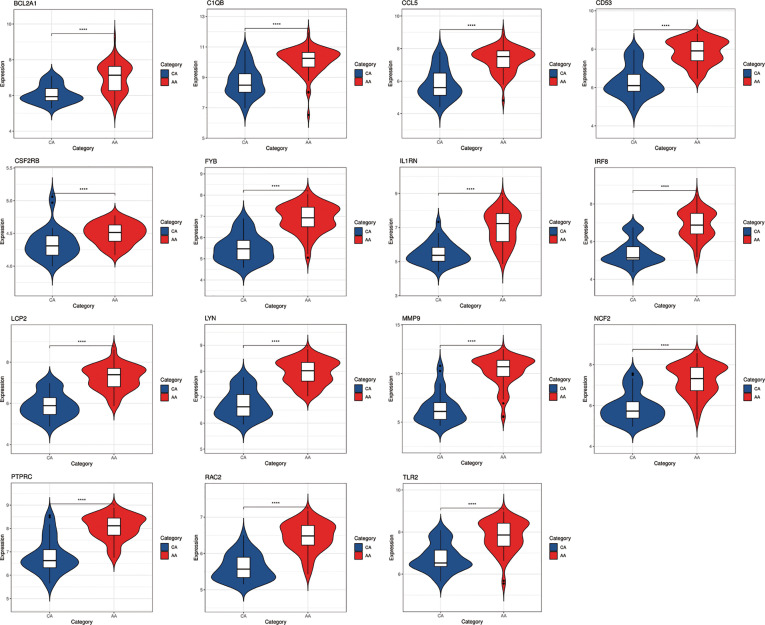
The expression level of hub gene in GSE100927. The comparison between the two sets of data uses the mean T test. P-value < 0.05 was considered statistically significant. AA, advanced atherosclerotic plaque; CA, control artery samples. ****p < 0.0001.

In order to verify the reliability of these hub gene expression levels. We selected two other data sets containing psoriasis lesions and atherosclerotic plaques and analyzed the expression levels of these hub genes. The results showed that compared with normal skin and normal tissue adjacent to the skin lesion, all hub genes were significantly up-regulated in psoriatic skin lesions (**Figure 7**). Similarly, the expression of all genes (the expression value of PLEK missing in GSE100927) in atherosclerotic plaques was also higher than in normal vascular tissues (**Figure 8**).

### Prediction and Verification of TFs

**Figure 9:**
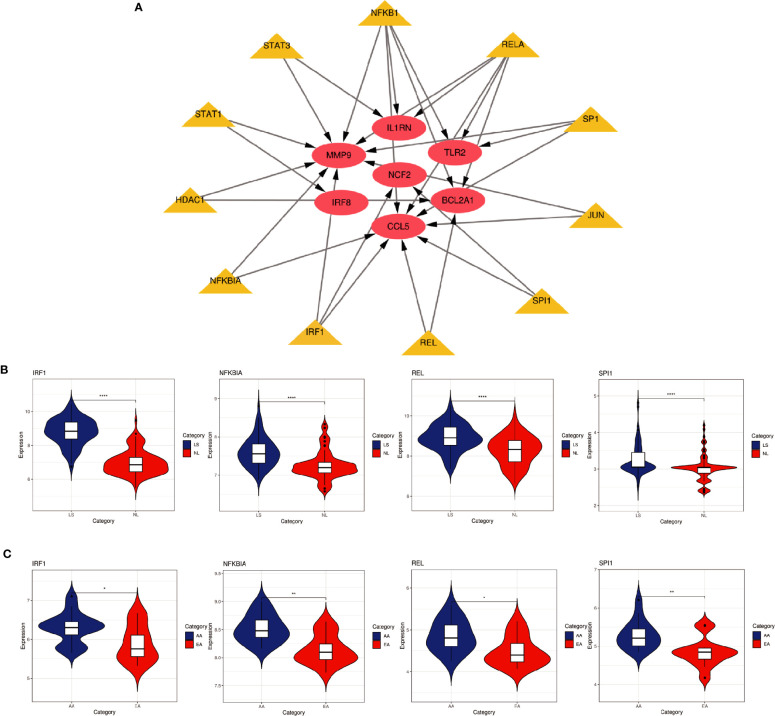
TFs regulatory network and its expression in GSE30999 and GSE28829. **(A)** TFs regulatory network. TFs were marked in yellow, and the hub genes were marked in red. **(B, C)** The expression level of TFs in GSE30999 and GSE28829. The comparison between the two sets of data uses the mean T test. P-value < 0.05 was considered statistically significant. LS, skin lesions; NL, adjacent normal tissues; AA, advanced atherosclerotic plaque; EA, early atherosclerotic plaque samples. *p < 0.05; **p < 0.01; ****p < 0.0001.

Based on the TRRUST database, we found that 11 TFs may regulate the expression of these genes (**Figure 9A** and **Table 3**). Further verification, we found that four TFs are highly expressed in psoriatic lesions and atherosclerotic plaques (**Figures 9B, C**). They coordinately participated in the regulation of four hub genes (CCL5, NCF2, MMP9 and BCL2A1).

**Table 3:** Key transcriptional factors (TFs) of hub genes.

Key TFs	Description	P-value	Genes
RELA	v-rel reticuloendotheliosis viral oncogene homolog A (avian)	2.14E-05	BCL2A1, MMP9, IL1RN, TLR2, CCL5
NFKB1	nuclear factor of kappa light polypeptide gene enhancer in B-cells 1	2.14E-05	CCL5, MMP9, BCL2A1, IL1RN, TLR2
IRF1	interferon regulatory factor 1	3.72E-05	CCL5, NCF2, MMP9
NFKBIA	nuclear factor of kappa light polypeptide gene enhancer in B-cells inhibitor, alpha	0.000314	MMP9, CCL5
REL	v-rel reticuloendotheliosis viral oncogene homolog (avian)	0.000339	BCL2A1, CCL5
SPI1	spleen focus forming virus (SFFV) proviral integration oncogene spi1	0.00227	NCF2, CCL5
HDAC1	histone deacetylase 1	0.00254	MMP9, BCL2A1
STAT1	signal transducer and activator of transcription 1, 91kDa	0.0031	MMP9, IRF8
STAT3	signal transducer and activator of transcription 3 (acute-phase response factor)	0.00691	IL1RN, MMP9
SP1	Sp1 transcription factor	0.00691	TLR2, MMP9, CCL5
JUN	jun proto-oncogene	0.00691	CCL5, MMP9

## Discussion

Insulin resistance induced by the inflammatory response in psoriasis can cause atherosclerosis. Research showed that a variety of inflammatory markers can be detected in the blood of patients with psoriasis, confirming that the body was in a systemic inflammatory state during the onset of psoriasis, which induced insulin resistance and reduced the release of vasodilator factors, then it provided an environmental basis for the formation of atherosclerotic plaques (19). Studies have shown that psoriasis is associated with atherosclerosis through dyslipidemia, including increased levels of total cholesterol, triglycerides, low-density lipoprotein, ultra-low-density lipoprotein and lipoprotein a, and the reduction of high-density lipoprotein and apolipoprotein B levels (20). Adipokines, including adiponectin, leptin, resistin, omentin, visfatin, chemoattractant and retinol binding protein 4, are considered to be risk factors for atherosclerosis. Among them, leptin, resistin, visfatin, chemoattractant and retinol binding protein 4 may be the common pathogenic factors of psoriasis and atherosclerosis. Adiponectin deficiency, leptin, visfatin and chemokine can induce and aggravate psoriasis by activating plasmacytoid dendritic cells and T cells (21, 22). It has been demonstrated that low-density granulocyte (LDG), which is a subset of neutrophils, is elevated in psoriasis and is related to skin disease severity and noncalcified coronary plaque burden. The main mechanism of neutrophil infiltration in patients with psoriasis complicated with atherosclerosis is that psoriasis LDGs may have a cytotoxic effect on endothelial cells, and in an inflammatory environment the adherence of LDG to platelets might be an important link between psoriasis skin disease severity and early atherogenesis (23).

Psoriasis and atherosclerosis might have overlapping pathogenic pathways. Inflammatory and immune regulatory pathways, such as IL23/IL17A pathways are linked to the pathogenesis of these two diseases. IL17A inhibitors are currently commonly used to treat psoriasis. The main purpose of our study is to identify the common DEGs in psoriasis and atherosclerosis. Thereby, revealing potential targets is to predict the therapeutic effect of biologics and treating psoriasis complicated with atherosclerosis.

We must acknowledge the genetic similarity between psoriasis and cardiovascular disease. Study found that single-nucleotide polymorphisms (SNPs) associated with increased risk for dyslipidemia (rs2247056, rs3177928, rs492602, and rs181362), increased blood pressure levels (rs805303, rs653178, and rs3184504), and increased risk for cardiovascular diseases (rs3184504) were associated with increased risk for psoriasis (24). The top three SNPs (rs2247056, rs3177928, and rs805303) were located in the HLA gene region. They also identified four non-HLA SNPs with evidence of shared genetic risk between psoriasis and cardiovascular and metabolic diseases: rs492603 in FUT2, rs181362 in UBE2L3, and rs653178 and rs3184504 near or in SH2B3 (24).

In this study, we identified 94 overlapping DEGs in both, of which 16 were hub genes, including LYN, CSF2RB, IL1RN, RAC2, CCL5, IRF8, C1QB, MMP9, PLEK, PTPRC, FYB, BCL2A1, LCP2, CD53, NCF2 and TLR2. GO and KEGG Pathway enrichment analysis revealed that these genes were significantly enriched in inflammatory and immune pathways. Chemokines and cytokines are jointly involved in the occurrence and development of these two inflammatory diseases, such as serum TNF-α, vascular endothelial growth factor, IL12, monocyte chemotaxis Protein 1, S100A8/A9 and significantly increased IL17A, which play a central role in the development of psoriasis and atherosclerosis. According to GO analysis, the lipopolysaccharide-mediated signaling pathway also play an important role in these two diseases. In psoriasis, lipopolysaccharide mediates nuclear transduction of NF-κB, then proinflammatory factors including TNF-α, IL6, and prostaglandin E (PGE) up- regulate in keratinocytes (25). In atherosclerosis, lipopolysaccharide, a Gram-negative endotoxin and an exogenous ligand for TLR4, can induce the activation of TLR4-MyD88-NF-κB signaling, followed by the release of atherosclerosis-related inflammatory factors such as TNF-α, IL1β, IL6, and MCP-1, leading to the development and progression of atherosclerosis (26). In addition, we found that 11 TFs may regulate the expression of these genes. By further verification, we found that four TFs are highly expressed in psoriatic lesions and atherosclerotic plaques, including IRF1, NFKBIA, REL, SPI1. They coordinately participated in the regulation of four hub genes (MMP9, CCL5, NCF2, and BCL2A1).

MMP9 is a member of MMPs, which endoproteases with collagenase and/or gelatinase activity which exert deleterious effects on the endothelium integrity and collagen fibers, promoting atherosclerotic plaque destabilization and accelerating the process of atherothrombosis (27, 28). Visfatin, which is the common pathogenic factors of psoriasis and atherosclerosis, induces the increased expression of MMP9 in the carotid plaques in an indirect way (29). Studies have confirmed MMP9 is overexpressed in psoriatic neutrophils, and detected to be abundant in psoriatic skin lesions, especially adjacent to endovascular and perivascular tissues, indicating MMP9 may play a role in vascular remodeling in psoriasis. In psoriasis, MMP9 also significantly upregulates the mRNA levels of ICAM-1, IL1β and CXCL1 in vascular endothelial cells (VECs), which enables VECs interaction with more leukocytes (30). CCL5 is expressed and secreted by normal T cell. It is reported that CCL5 production by keratinocytes is increased in psoriatic skin lesions. CCL5 may contribute to the T helper-1-dominant infiltration in psoriatic lesions as well as CXC-chemokines. CCL5 also recruits macrophages. T helper-1 cells or macrophages infiltrating into psoriatic lesions in turn may produce CCL5 and proinflammatory cytokines such as TNF-α, IL1β, or INF-γ, which promote CCL5 production by keratinocytes. Thus, CCL5 production in psoriatic lesions may amplify the inflammation in the lesions (31). In atherosclerosis, macrophages are the major CCL5-expressing cells in the atherosclerotic plaque, CCL5 expression in macrophages is promoted by IFN-γ. CD8α^+^ dendritic cells aggravate atherosclerosis, likely by inducing Th1 cell response, which promotes CCL5 expression in macrophages and increased infiltration of leukocytes and lesion inflammation (32). BCL2A1 is a member of the BCL2 family. The process of apoptosis is controlled by proteins of the BCL2 family, Increased expression of BCL2A1 protein was associated with psoriatic epidermal hyperplasia (33). It’s also involved in vascular endothelial cell apoptosis and the formation of atherosclerotic plaque. Macrophage resistance to apoptosis was enhanced using macrophage-specific human BCL2 overexpression, have also shown accelerated early atherosclerotic plaque development (34). NCF2 gene encodes neutrophil cytosolic factor 2, the 67-kilodalton cytosolic subunit of the multi-protein complex, which is known as nicotinamide-adenine dinucleotide phosphate (NADPH) oxidase found in neutrophils. This oxidase produces a burst of superoxide which is delivered to the lumen of the neutrophil phagosome (35). As the main source of reactive oxygen species (ROS), the NADPH oxidase family plays an important role in various cellular functions such as antibacterial, anti-inflammatory, and redox signaling (36). ROS are messenger molecules that play a prominent role in cell metabolism-regulating cell proliferation, differentiation and death (37). In psoriasis, it has been reported that an increased intracellular ROS production and an enhanced NADPH oxidase activity in fibroblasts obtained from skin lesions of psoriatic patients (38). NADPH oxidase 4 expressed in dermal fibroblasts is essential for the redox paracrine regulation of epidermal keratinocytes proliferation (39). In atherosclerosis, oxidative stress is a risk factor for it. NADPH seems to mediate its detrimental effects by promoting inflammation in atherosclerosis (40). However, few studies directly analyze the role of NCF2 in psoriasis and atherosclerosis, which emphasizes its importance in future research.

Prior study explored the inflammatory and lipid genes related to atherosclerotic cardiovascular disease in skin and serum from patients with psoriasis (41). It found an increase in skin and serum gene expression for MCP-1 and MDC in psoriasis compared with healthy controls, and lipid metabolism (LXR-α, PPAR-α) genes decreased in psoriasis versus healthy controls (41). Our study also found that new genes like CSF2RB, IL1RN, CCL5, MMP9, CD53, NCF2 and TLR2 associated with Inflammation present high expression both in psoriasis and atherosclerosis. In addition, the lipopolysaccharide-mediated signaling pathway which was found involving many co-expressed genes would induce the release of MCP-1.

Mehta et al. used a bioinformatics approach of lesional psoriasis skin and atherosclerotic plaques, showed that IFN-γ and TNF-α are the dominant pro-inflammatory signals linking atherosclerosis and psoriasis, more importantly, both IFN-γ and TNF-α are increased in the serum of patients with moderate-to-severe psoriasis and their respective receptors in atherosclerotic plaques are increased. Stimulation of primary aortic endothelial cells and ex-vivo atherosclerotic tissue with IFN-γ and TNF-α synergistically increased monocyte and T-cell chemoattractants and adhesion molecules, concomitant with a decrease in endothelial barrier integrity. Their finding suggested IFN-γ/TNF-α synergy may provide a critical pro-inflammatory link between psoriatic skin inflammation and distant vessel atherosclerosis (42). Because of the strong genetic similarity between psoriasis and cardiovascular diseases (24), unlike previous studies, our research pays more attention to the exploration of hub genes and related TFs that are common to psoriasis and atherosclerosis. We have constructed a complex interaction network to identify the key nodes through their common DEGs. This comprehensive bioinformatics method has been proven to be reliable in a variety of diseases (43, 44). In addition, we also analyzed related TFs and verified their expression levels in the original data set. Our research will provide potential directions for the study of the molecular mechanism of psoriasis complicated by atherosclerosis.

Although previous studies have separately explored the hub genes associated with psoriasis (45) and atherosclerosis (46). However, few studies have explored the common molecular mechanism between them by advanced bioinformatics methods. Due to the high comorbidity rate between psoriasis and atherosclerosis, we explored and identified the common DEGs, hub genes and TFs between the two for the first time, which helped to further clarify the mechanism of psoriasis and atherosclerosis. However, our research also has some limitations. First of all, this is a retrospective study that requires external verification to verify our findings; Secondly, the function of the hub gene needs to be further verified in an *in vitro* model, which will be the focus of our future work.

## Conclusions

In summary, we identified the common DEGs of psoriasis and atherosclerosis and performed enrichment and PPI network analysis. We found that psoriasis and atherosclerosis had many common pathogenic mechanisms, which may be mediated by specific hub genes. This study provides new insights for the further study of the molecular mechanism of psoriasis complicated with atherosclerosis.

## Data Availability Statement

The original contributions presented in the study are included in the article/**Supplementary Material**. Further inquiries can be directed to the corresponding authors.

## Author Contributions

JJ and YS developed a major research plan. WS and YZ analyze data, draw charts and write manuscripts. XZ and YW helped collect data and references. All authors contributed to the article and approved the submitted version.

## Funding

This work was supported by Suzhou Minsheng Technology - Medical and Health Application Foundation (SYS2020135) and 2020 Medical Research Project of Jiangsu Provincial Health Commission (Z2020017).

## Conflict of Interest

The authors declare that the research was conducted in the absence of any commercial or financial relationships that could be construed as a potential conflict of interest.

## Supplementary Material

Supplementary Table 1 The details of the common DEGs.

## Abbreviations

GEO, Gene Expression Omnibus; DEGs, differentially expressed genes; PPI, protein‐protein interaction; LS: skin lesions; NL, adjacent normal tissues; EA, early atherosclerotic plaque samples; AA, advanced atherosclerotic plaque samples; GO, Gene ontology; KEGG, Kyoto Encyclopedia of Genes and Genomes; KOBAS, KEGG Orthology Based Annotation System; MCODE, molecular complex detection technology; NS, normal skin; CA, control artery samples; TFs, transcription factors; TRRUST, Transcriptional Regulatory Relationships Unraveled by Sentence-based Text mining; LDG, low-density granulocyte; SNP, single-nucleotide polymorphism; PGE, prostaglandin E; VECs, vascular endothelial cells vascular endothelial cells; ROS, reactive oxygen species; NADPH, nicotinamide-adenine dinucleotide phosphate.

## References

[1] Lockshin B Balagula Y Merola J. Interleukin 17, Inflammation, and Cardiovascular Risk in Patients With Psoriasis. J Am Acad Dermatol (2018) 79(2):345–52. 10.1016/j.jaad.2018.02.040 29477740

[2] Armstrong A Harskamp C Ledo L Rogers J Armstrong E. Coronary Artery Disease in Patients With Psoriasis Referred for Coronary Angiography. Am J Cardiol (2012) 109(7):976–80. 10.1016/j.amjcard.2011.11.025 22221950

[3] Golden J McCormick T Ward N. Il-17 in Psoriasis: Implications for Therapy and Cardiovascular Co-Morbidities. Cytokine (2013) 62(2):195–201. 10.1016/j.cyto.2013.03.013 23562549 PMC3640599

[4] Forkel S Schön M Hildmann A Claßen A John S Danker K. Inositoylated Platelet-Activating Factor (Ino-C2-PAF) Modulates Dynamic Lymphocyte-Endothelial Cell Interactions and Alleviates Psoriasis-Like Skin Inflammation in Two Complementary Mouse Models. J Invest Dermatol (2014) 134(10):2510–20. 10.1038/jid.2014.170 24714204

[5] Liu T Li S Ying S Tang S Ding Y Li Y. The IL-23/IL-17 Pathway in Inflammatory Skin Diseases: From Bench to Bedside. Front Immunol (2020) 11:594735. 10.3389/fimmu.2020.594735 33281823 PMC7705238

[6] Wolf D Ley K. Immunity and Inflammation in Atherosclerosis. Circ Res (2019) 124(2):315–27. 10.1161/circresaha.118.313591 PMC6342482 30653442

[7] Taleb S Tedgui A. Il-17 in Atherosclerosis: The Good and the Bad. Cardiovasc Res (2018) 114(1):7–9. 10.1093/cvr/cvx225 29228116

[8] Edgar R Domrachev M Lash AE. Gene Expression Omnibus: NCBI Gene Expression and Hybridization Array Data Repository. Nucleic Acids Res (2002) 30(1):207–10. 10.1093/nar/30.1.207 PMC99122 11752295

[9] Suárez-Fariñas M Li K Fuentes-Duculan J Hayden K Brodmerkel C Krueger J. Expanding the Psoriasis Disease Profile: Interrogation of the Skin and Serum of Patients With Moderate-to-Severe Psoriasis. J Invest Dermatol (2012) 132(11):2552–64. 10.1038/jid.2012.184 PMC3472561 22763790

[10] Döring Y Manthey H Drechsler M Lievens D Megens R Soehnlein O. Auto-Antigenic Protein-DNA Complexes Stimulate Plasmacytoid Dendritic Cells to Promote Atherosclerosis. Circulation (2012) 125(13):1673–83. 10.1161/circulationaha.111.046755 22388324

[11] Barrett T Wilhite SE Ledoux P Evangelista C Kim IF Tomashevsky M. Ncbi GEO: Archive for Functional Genomics Data Sets–Update. Nucleic Acids Res (2013) 41(Database issue):D991–5. 10.1093/nar/gks1193 PMC3531084 23193258

[12] Wu J Mao X Cai T Luo J Wei L. KOBAS Server: A Web-Based Platform for Automated Annotation and Pathway Identification. Nucleic Acids Res (2006) 34(Web Server Issue):W720–4. 10.1093/nar/gkl167 PMC1538915 16845106

[13] Franceschini A Szklarczyk D Frankild S Kuhn M Simonovic M Roth A. String v9.1: Protein-Protein Interaction Networks, With Increased Coverage and Integration. Nucleic Acids Res (2013) 41(Database issue):D808–15. 10.1093/nar/gks1094 PMC3531103 23203871

[14] Smoot ME Ono K Ruscheinski J Wang PL Ideker T. Cytoscape 2.8: New Features for Data Integration and Network Visualization. Bioinformatics (2011) 27(3):431–2. 10.1093/bioinformatics/btq675 PMC3031041 21149340

[15] Warde-Farley D Donaldson SL Comes O Zuberi K Badrawi R Chao P. The GeneMANIA Prediction Server: Biological Network Integration for Gene Prioritization and Predicting Gene Function. Nucleic Acids Res (2010) 38(Web Server Issue):W214–20. 10.1093/nar/gkq537 PMC2896186 20576703

[16] Yao Y Richman L Morehouse C de los Reyes M Higgs B Boutrin A. Type I Interferon: Potential Therapeutic Target for Psoriasis? PloS One (2008) 3(7):e2737. 10.1371/journal.pone.0002737 18648529 PMC2481274

[17] Steenman M Espitia O Maurel B Guyomarch B Heymann M Pistorius M. Identification of Genomic Differences Among Peripheral Arterial Beds in Atherosclerotic and Healthy Arteries. Sci Rep (2018) 8(1):3940. 10.1038/s41598-018-22292-y 29500419 PMC5834518

[18] Han H Cho JW Lee S Yun A Kim H Bae D. Trrust v2: An Expanded Reference Database of Human and Mouse Transcriptional Regulatory Interactions. Nucleic Acids Res (2018) 46(D1):D380–d386. 10.1093/nar/gkx1013 29087512 PMC5753191

[19] Gonzalez-Cantero A Gonzalez-Cantero J Sanchez-Moya A Perez-Hortet C Arias-Santiago S Schoendorff-Ortega C. Subclinical Atherosclerosis in Psoriasis. Usefulness of Femoral Artery Ultrasound for the Diagnosis, and Analysis of Its Relationship With Insulin Resistance. PLoS One (2019) 14(2):e0211808. 10.1371/journal.pone.0211808 30735527 PMC6368294

[20] Praveenkumar U Ganguly S Ray L Nanda S Kuruvila S. Prevalence of Metabolic Syndrome in Psoriasis Patients and Its Relation to Disease Duration: A Hospital Based Case-Control Study. J Clin Diagn Res JCDR (2016) 10(2):WC01–5. 10.7860/jcdr/2016/17791.7218 PMC4800631 27042565

[21] Shibata S Tada Y Hau C Mitsui A Kamata M Asano Y. Adiponectin Regulates Psoriasiform Skin Inflammation by Suppressing IL-17 Production From γδ-T Cells. Nat Commun (2015) 6:7687. 10.1038/ncomms8687 26173479

[22] Baran A Flisiak I Jaroszewicz J Świderska M. Effect of Psoriasis Activity on Serum Adiponectin and Leptin Levels. Postepy Dermatol i Alergol (2015) 32(2):101–6. 10.5114/pdia.2014.40960 PMC4436231 26015779

[23] Teague H Varghese N Tsoi L Dey A Garshick M Silverman J. Neutrophil Subsets, Platelets, and Vascular Disease in Psoriasis. JACC Basic to Trans Sci (2019) 4(1):1–14. 10.1016/j.jacbts.2018.10.008 PMC6390681 30847414

[24] Lu Y Chen H Nikamo P Qi Low H Helms C Seielstad M. Association of Cardiovascular and Metabolic Disease Genes With Psoriasis. J Invest Dermatol (2013) 133(3):836–9. 10.1038/jid.2012.366 PMC3570714 23190900

[25] Donnarumma G Paoletti I Buommino E Fusco A Baudouin C Msika P. AV119, a Natural Sugar From Avocado Gratissima, Modulates the LPS-Induced Proinflammatory Response in Human Keratinocytes. Inflammation (2011) 34(6):568–75. 10.1007/s10753-010-9264-6 20936426

[26] Kapelouzou A Giaglis S Peroulis M Katsimpoulas M Moustardas P Aravanis C. Overexpression of Toll-Like Receptors 2, 3, 4, and 8 Is Correlated to the Vascular Atherosclerotic Process in the Hyperlipidemic Rabbit Model: The Effect of Statin Treatment. J Vasc Res (2017) 54(3):156–69. 10.1159/000457797 28478461

[27] Cowan K Jones P Rabinovitch M. Elastase and Matrix Metalloproteinase Inhibitors Induce Regression, and Tenascin-C Antisense Prevents Progression, of Vascular Disease. J Clin Invest (2000) 105(1):21–34. 10.1172/jci6539 10619858 PMC382582

[28] Shi Y Patel S Niculescu R Chung W Desrochers P Zalewski A. Role of Matrix Metalloproteinases and Their Tissue Inhibitors in the Regulation of Coronary Cell Migration. Arterioscler Thromb Vasc Biol (1999) 19(5):1150–5. 10.1161/01.atv.19.5.1150 10323763

[29] Li B Zhao Y Liu H Meng B Wang J Qi T. Visfatin Destabilizes Atherosclerotic Plaques in Apolipoprotein E-Deficient Mice. PLoS One (2016) 11(2):e0148273. 10.1371/journal.pone.0148273 26848572 PMC4743838

[30] Chen J Zhu Z Li Q Lin Y Dang E Meng H. Neutrophils Enhance Cutaneous Vascular Dilation and Permeability to Aggravate Psoriasis by Releasing Matrix Metallopeptidase 9. J Invest Dermatol (2020) 141(4):787–99. 10.1016/j.jid.2020.07.028 32888954

[31] Kanda N Watanabe S. 17beta-Estradiol Inhibits the Production of RANTES in Human Keratinocytes. J Invest Dermatol (2003) 120(3):420–7. 10.1046/j.1523-1747.2003.12067.x 12603855

[32] Li Y Liu X Duan W Tian H Zhu G He H. Batf3-dependent Cd8α Dendritic Cells Aggravates Atherosclerosis Via Th1 Cell Induction and Enhanced CCL5 Expression in Plaque Macrophages. EBioMedicine (2017) 18:188–98. 10.1016/j.ebiom.2017.04.008 PMC5405198 28411140

[33] Batinac T Zamolo G Hadzisejdić I Zauhar G Brumini G Ruzić A. Expression of Bcl-2 Family Proteins in Psoriasis. Croatian Med J (2007) 48(3):319–26. PMC2080542 17589974

[34] Shearn A Deswaerte V Gautier E Saint-Charles F Pirault J Bouchareychas L. Bcl-X Inactivation in Macrophages Accelerates Progression of Advanced Atherosclerotic Lesions in Apoe(-/-) Mice. Arterioscler Thromb Vasc Biol (2012) 32(5):1142–9. 10.1161/atvbaha.111.239111 22383704

[35] Martner A Aydin E Hellstrand K. NOX2 in Autoimmunity, Tumor Growth and Metastasis. J Pathol (2019) 247(2):151–4. 10.1002/path.5175 PMC6587556 30270440

[36] Breitenbach M Rinnerthaler M Weber M Breitenbach-Koller H Karl T Cullen P. The Defense and Signaling Role of NADPH Oxidases in Eukaryotic Cells: Review. Wiener Med Wochenschr (1946) (2018) 168:286–99. 10.1007/s10354-018-0640-4 PMC6132560 30084091

[37] Lisse T King B Rieger S. Comparative Transcriptomic Profiling of Hydrogen Peroxide Signaling Networks in Zebrafish and Human Keratinocytes: Implications Toward Conservation, Migration and Wound Healing. Sci Rep (2016) 6:20328. 10.1038/srep20328 26846883 PMC4742856

[38] Barygina V Becatti M Lotti T Taddei N Fiorillo C. Low Dose Cytokines Reduce Oxidative Stress in Primary Lesional Fibroblasts Obtained From Psoriatic Patients. J Dermatol Sci (2016) 83(3):242–4. 10.1016/j.jdermsci.2016.06.002 27317477

[39] Barygina V Becatti M Prignano F Lotti T Taddei N Fiorillo C. Fibroblasts to Keratinocytes Redox Signaling: The Possible Role of ROS in Psoriatic Plaque Formation. Antioxid (Basel Switzerland) (2019) 8(11):566. 10.3390/antiox8110566 PMC6912201 31752190

[40] Marqués J Cortés A Pejenaute Á Zalba G. Implications of NADPH Oxidase 5 in Vascular Diseases. Int J Biochem Cell Biol (2020) 128:105851. 10.1016/j.biocel.2020.105851 32949687

[41] Mehta N Li K Szapary P Krueger J Brodmerkel C. Modulation of Cardiometabolic Pathways in Skin and Serum From Patients With Psoriasis. J Trans Med (2013) 11:194. 10.1186/1479-5876-11-194 PMC3765699 23965158

[42] Mehta N Teague H Swindell W Baumer Y Ward N Xing X. Ifn-γ and TNF-α Synergism may Provide a Link Between Psoriasis and Inflammatory Atherogenesis. Sci Rep (2017) 7(1):13831. 10.1038/s41598-017-14365-1 29062018 PMC5653789

[43] Fang X Duan S Gong Y Wang F Chen X. Identification of Key Genes Associated With Changes in the Host Response to Severe Burn Shock: A Bioinformatics Analysis With Data From the Gene Expression Omnibus (Geo) Database. J Inflammation Res (2020) 13:1029–41. 10.2147/jir.S282722 PMC7718973 33293847

[44] Yang S Cao C Xie Z Zhou Z. Analysis of Potential Hub Genes Involved in the Pathogenesis of Chinese Type 1 Diabetic Patients. Ann Trans Med (2020) 8(6):295. 10.21037/atm.2020.02.171 PMC7186604 32355739

[45] Gao L Shen J Ren Y Shi J Wang D Cao J. Discovering Novel Hub Genes and Pathways Associated With the Pathogenesis of Psoriasis. Dermatol Ther (2020) 33(6):e13993. 10.1111/dth.13993 32648291

[46] Chen P Chen Y Wu W Chen L Yang X Zhang S. Identification and Validation of Four Hub Genes Involved in the Plaque Deterioration of Atherosclerosis. Aging (2019) 11(16):6469–89. 10.18632/aging.102200 PMC6738408 31449494

