# Supplementary material for: Exploring the Pathogenesis of Psoriasis Complicated With Atherosclerosis via Microarray Data Analysis
Source: Front Immunol. 2021 May 27;12:667690. doi: 10.3389/fimmu.2021.667690 (PMC8190392; doi:10.3389/fimmu.2021.667690)
Supplement: Supplementary Table 1 — The details of the common DEGs. [file Table_1.docx]

| Gene symbol | Expression | GSE30999  Log2(FC) | GSE28829  Log2(FC) |
| --- | --- | --- | --- |
| ATP1A2 | down | -2.44 | -1.83392 |
| PDE4DIP | down | -2.08 | -1.01295 |
| ACADL | down | -1.43 | -1.36529 |
| ZEB1 | down | -1.08 | -1.02641 |
| KCNMA1 | down | -1.03 | -1.05887 |
| MYOCD | down | -1.79 | -1.02727 |
| FAT3 | down | -1.51 | -1.03246 |
| FIBIN | down | -1.75 | -1.00725 |
| CAB39L | down | -1.49 | -1.05567 |
| RBP4 | down | -2.43 | -1.11497 |
| SBSPON | down | -1.33 | -1.01643 |
| SH3BGR | down | -1.55 | -1.17032 |
| SCUBE3 | down | -1.71 | -1.04991 |
| BTC | down | -4.75 | -1.71674 |
| ANGPTL1 | down | -1.14 | -1.15016 |
| C3orf70 | down | -1.12 | -1.35358 |
| CNTN4 | down | -1.4 | -1.27108 |
| PPP1R1A | down | -2.37 | -1.12101 |
| CASQ2 | down | -2.06 | -1.16446 |
| TPH1 | down | -1.1 | -1.09299 |
| CCDC146 | down | -1.6 | -1.06311 |
| PRUNE2 | down | -1.22 | -1.08905 |
| NTN1 | down | -1.04 | -1.0226 |
| NEXN | down | -1.51 | -1.05581 |
| CYTIP | up | 1.33 | 1.420812 |
| IL7R | up | 2.34 | 1.026407 |
| LYN | up | 2.51 | 1.147452 |
| POU2AF1 | up | 1.05 | 1.116756 |
| DOK3 | up | 2.11 | 1.035259 |
| MS4A7 | up | 1.4 | 1.684681 |
| FABP5 | up | 1.47 | 1.654148 |
| DENND2D | up | 1.07 | 1.154652 |
| CCL8 | up | 1.88 | 1.141896 |
| LCP2 | up | 1.24 | 1.200911 |
| ACP5 | up | 1.75 | 2.052267 |
| CD53 | up | 1.07 | 1.66447 |
| GPR183 | up | 1.88 | 1.125223 |
| KYNU | up | 7.27 | 2.036197 |
| ABCG1 | up | 1.3 | 1.165426 |
| RALA | up | 1.64 | 1.097317 |
| SLAMF8 | up | 1.19 | 2.072546 |
| ADAP2 | up | 3.41 | 1.223717 |
| AMPD3 | up | 1.52 | 1.253758 |
| RAC2 | up | 1.21 | 1.547076 |
| CCL5 | up | 1.27 | 1.087311 |
| CD36 | up | 1.22 | 1.737681 |
| CEMIP | up | 2.19 | 1.711369 |
| SERPINA1 | up | 4.23 | 1.732502 |
| C1QB | up | 1.29 | 1.905494 |
| FAM26F | up | 2.83 | 1.002991 |
| PYCARD | up | 1.42 | 1.100445 |
| ADAMDEC1 | up | 5.11 | 1.790156 |
| SEL1L3 | up | 1.09 | 1.404221 |
| CTSB | up | 2 | 1.310966 |
| NCF2 | up | 1.36 | 1.537785 |
| TLR2 | up | 1.99 | 1.0731 |
| SNX10 | up | 1.35 | 1.209333 |
| C15orf48 | up | 2.15 | 1.364491 |
| SLC2A5 | up | 1.3 | 1.061119 |
| MMP12 | up | 3.96 | 2.576197 |
| HK2 | up | 1.07 | 1.12515 |
| RRM2 | up | 4.99 | 1.174333 |
| PAG1 | up | 1.35 | 1.08631 |
| CARD16 | up | 1.12 | 1.12324 |
| CSF2RB | up | 1.06 | 1.634016 |
| CXCL2 | up | 3.96 | 2.034357 |
| KIAA0101 | up | 2.47 | 1.30861 |
| IRF8 | up | 1.08 | 1.127065 |
| GALNT6 | up | 3.64 | 1.028412 |
| CD48 | up | 1.61 | 1.323418 |
| SLC16A10 | up | 2.6 | 1.590091 |
| FYB | up | 1.42 | 1.376456 |
| BCL2A1 | up | 3.78 | 1.404463 |
| RGS1 | up | 4.01 | 1.485072 |
| CARD10 | up | 1.21 | 1.060183 |
| CCL18 | up | 2.36 | 2.564887 |
| CD83 | up | 2.29 | 1.061795 |
| FAM20A | up | 1.28 | 1.028985 |
| FPR3 | up | 1.52 | 1.527008 |
| SOCS3 | up | 1.31 | 1.220629 |
| IL1RN | up | 1.37 | 1.272575 |
| CHI3L2 | up | 3.88 | 1.459566 |
| NPL | up | 1.54 | 1.916589 |
| MMP9 | up | 1.45 | 2.01113 |
| JAK3 | up | 1.34 | 1.090396 |
| LINC01094 | up | 3.52 | 1.104971 |
| PLEK | up | 1.72 | 1.35109 |
| PTPRC | up | 1.06 | 1.559453 |
| ST8SIA4 | up | 1.34 | 1.11706 |
| F11R | up | 1.79 | 1.043014 |
| CTSC | up | 1.25 | 1.299114 |
| CLEC5A | up | 1.49 | 1.539905 |
| PLAUR | up | 1.6 | 1.069984 |
| ITGA4 | up | 1.45 | 1.275812 |
